# Clinical Theileriosis in an Endangered Malayan Tapir Diagnosed Using a Molecular Technique with Successful Treatment and Recovery

**DOI:** 10.3390/ani15213131

**Published:** 2025-10-29

**Authors:** Pornsuda Khotapat, Umaporn Maikaew, Wanlaya Tipkantha, Erngsiri Kaewkhunjob, Piyaporn Kongmakee, Arpussara Saedan, Pannarai Mahadthai, Manakorn Sukmak, Supaphen Sripiboon

**Affiliations:** 1 Animal Conservation and Research Institute, The Zoological Park Organization of Thailand, Dusit, Bangkok 10300, Thailand; pornsuda.kho@zoothailand.org (P.K.); wanlayav62@gmail.com (W.T.); erngsiri.k@gmail.com (E.K.); arpussara.s@gmail.com (A.S.); pm.ks1502@gmail.com (P.M.); 2 Khao Kheow Open Zoo, Sri Racha, Chon Buri 20110, Thailand; au_elephant@hotmail.com; 3 Department of Farm Resources and Production Medicine, Faculty of Veterinary Medicine, Kasetsart University, Kamphaeng Saen, Nakhon Pathom 73140, Thailand; fvetmksu@gmaill.com; 4 Department of Large Animal and Wildlife Clinical Science, Faculty of Veterinary Medicine, Kasetsart University, Kamphaeng Saen, Nakhon Pathom 73140, Thailand

**Keywords:** 18S rRNA, tapir, *Theileria orientalis*, theileriosis, treatment

## Abstract

A 12-year-old female captive Malayan tapir (*Tapirus indicus*) in Thailand presented with clinical signs including depression, anorexia, pale mucous membrane, dark-brown urine, dry feces, and tick infestation. Hematological analysis revealed severe anemia (packed cell volume, PCV = 9%), and blood smear examination suggested blood parasite infection, which was subsequently confirmed as *Theileria orientalis* using PCR targeting the 18S rRNA gene and sequencing. Treatment consisted of the administration of diminazene aceturate (2.5 mg/kg, IM, q14days for three doses), whole-blood transfusion, fluid therapy, and other supportive care. Clinical improvement was observed within three days post-treatment, including normalization of urine color and a progressive increase in PCV. By Day 42, the PCV had returned to normal (33%), with no observed complications from the medication or blood transfusion. Further screening using PCR targeting the 18S rRNA gene of piroplasmid parasites revealed an 80% apparent prevalence of *Theileria* infection among sampled tapirs in the same facility, although only one individual exhibited clinical signs. These findings indicate that asymptomatic carriage of *T. orientalis* can be observed in tapirs and that clinical disease could emerge under stress or immunocompromised conditions. Given the presence of ticks and potential contact with other susceptible species in zoo settings, routine surveillance and preventive tick control are essential for mitigating transmission risks.

## Simple Summary

This report describes the first confirmed case of clinical theileriosis (*Theileria orientalis*) in a captive Malayan tapir in Thailand. Although this pathogen is typically endemic in cattle in Asia, where it often causes asymptomatic infection, it can lead to severe clinical signs in immunocompromised animals. The affected tapir was successfully treated with an anti-protozoal drug and blood transfusion. This case highlights the importance of long-term management, routine monitoring, and effective tick control for vulnerable wildlife species.

## 1. Introduction

The Malayan tapir (*Tapirus indicus*), the largest of the four extant tapir species, belongs to the family Tapiridae within the order Perrissodactyla, commonly known as the odd-toed ungulates. This species is native to Southeast Asia, with a geographic range encompassing Thailand, Myanmar, Peninsular Malaysia, and Sumatra, Indonesia [1,2]. As a flagship species for tropical forest conservation in the region, the health and survival of the Malayan tapir are critical not only for its own conservation but also for the maintenance of broader ecosystem biodiversity. The Malayan tapir is currently classified as Endangered by the International Union for Conservation of Nature [3], primarily due to habitat loss, fragmentation, and low reproductive rates. Ex situ conservation efforts play a crucial role in safeguarding the species and maintaining its genetic diversity.

Among the factors that may impact tapir health, infectious diseases caused by hemoparasites are of increasing concern. *Theileria* spp. are obligate intracellular protozoan parasites belonging to the phylum Apicomplexa, order Piroplasmida, that infect a wide range of host species, particularly ungulates in tropical and subtropical regions [4,5]. These tick-borne hemoparasites, primarily transmitted by ticks, exhibit a complex life cycle, involving schizogony in lymphocytes and merogony in erythrocytes [6]. In addition, *Theileria* spp. are broadly categorized into two groups based on their ability to induce host leukocyte proliferation: transforming and non-transforming groups [7,8]. Most pathogenic species belong to the transforming group, which includes *T. parva* and *T. annulata*, whereas the non-transforming group (also referred to as benign *Theileria*), which includes *T. orientalis* and *T. mutans,* generally causes mild or asymptomatic infection [8,9]. Although the non-transforming group is typically considered less pathogenic, significant morbidity due to hemolytic anemia may occur, particularly in immunosuppressed or co-infected animals [7].

Wildlife can act as reservoirs for *Theileria* spp., contributing to the persistence and transmission of the pathogen across ecological boundaries. The presence of *Theileria* is of growing concern, not only due to its impact on livestock health and agricultural economies but also for its implications on wildlife conservation and management [10]. While natural *Theileria* infections have previously been reported in other tapir species, without clinical signs [11,12], no cases of clinical disease have previously been documented in Malayan tapirs. In this report, we describe the first clinical case of a non-transforming species, *T. orientalis*, in a captive Malayan tapir in Thailand, detailing the clinical presentation, treatment protocol, and outcome. Furthermore, we highlight that, due to the morphological similarity among piroplasm species, molecular testing targeting the 18S rRNA gene was used for species identification.

## 2. Case Presentation

On 1 November 2021 (Day 1), a 12-year-old captive female Malayan tapir, estimated to weigh 250 kg, was observed to have reduced appetite. In addition, only a small amount of dry feces was found in her holding area. At the time, she was nursing a 2-month-old calf. A broad-spectrum antibiotic (Enrofloxacin 5 mg/kg) and non-steroidal anti-inflammatory drug (Flunixin meglumine 1 mg/kg) were administered intramuscularly. On the following day (Day 2), anorexia persisted, and the zookeeper reported additional clinical signs, including lethargy, constipation, and dark-brown urine.

**Figure 1:**
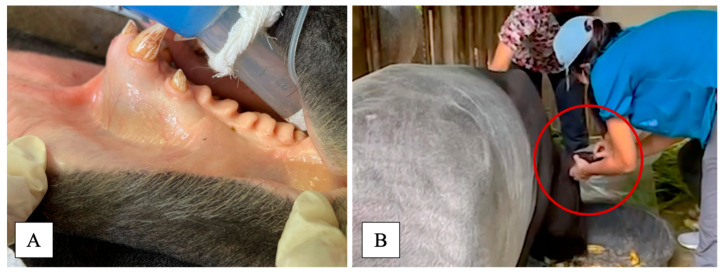
Physical examination revealed pale mucous membrane (**A**); blood was collected from ear vein (red circle) using positive reinforcement without sedation/anesthesia (**B**).

As the tapir had previously undergone target training through positive reinforcement, an initial physical examination could be performed without sedation or anesthesia. The initial physical examination revealed tachypnea, pale mucous membrane (Figure 1A), and the presence of ticks on both ears and the dorsal body. Blood samples were also obtained from the ear vein in EDTA-coated and plain tubes using positive reinforcement (Figure 1B).

**Figure 2:**
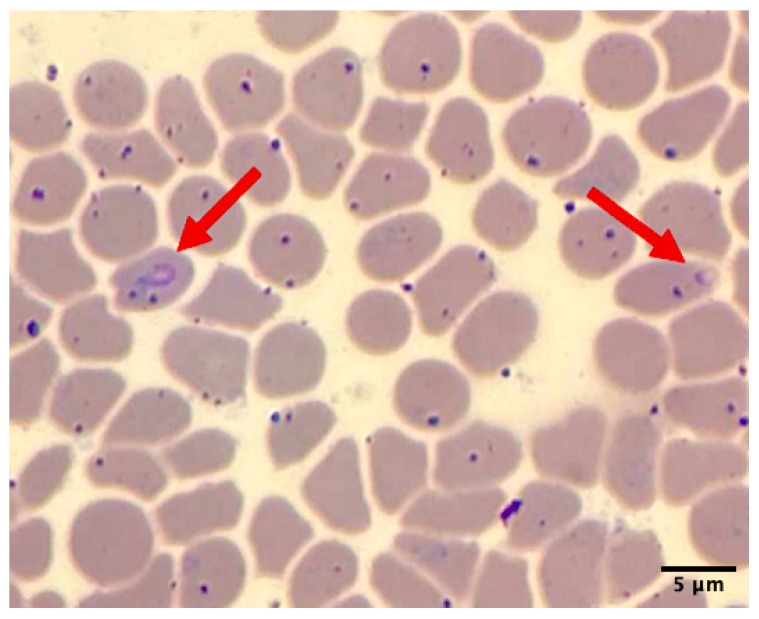
Thin blood smear of sample on Day 2 showed piroplasm-like structure on erythrocytes (red arrow) under microscopic examination (100X).

Hemolysis and icteric serum were observed in the blood samples from this animal. Hematological results indicated severe normochromic normocytic anemia with a decreased packed cell volume (9%; normal 31.4–36.4), hemoglobin level (3.5 g/dL; normal 11.0–13.0), and total red blood cell count (1.48 × 10^6^ cells/μL; normal 4.6–6.0 × 10^6^); thrombocytopenia (45,000 cells/μL; normal 231,800–550,400); and leukopenia (4.8 × 10^3^ cells/μL; normal 6.7–9.7 × 10^3^). The serum chemistry levels showed hypoproteinemia (5.0 g/dL; normal 6.3–7.1), an elevated AST level (258 IU/L; normal 96.2–2222.6), an increased CPK level (1362 IU/L; normal 175–328), and hyperbilirubinemia (3.2 mg/dL; normal 0.23–0.55). Additionally, a thin peripheral blood smear stained with Wright–Giemsa revealed an intraerythrocytic piroplasm-like structure under light microscopic examination (Figure 2).

Based on the physical examination and laboratory findings, the veterinary team suspected a piroplasmid infection as the primary cause of the tapir’s illness. On the same day (Day 2), initial treatment was initiated with 630 mg of diminazene aceturate (Berenil®, MSD Animal Health, Pune, India; 2.5 mg/kg, intramuscularly). Given the severity of the anemia, a blood transfusion was also planned to address this life-threatening condition. Five healthy adult male Malayan tapirs from the same collection were evaluated as potential blood donors based on the results of major blood cross-matching tests, complete blood counts, and logistical considerations regarding their proximity to the recipient. All candidates exhibited hematological parameters within the normal range, showed no detectable blood parasites on microscopic examination, and demonstrated no hemolytic reactions during the cross-matching test. A 4-year-old male tapir weighing approximately 300 kg was ultimately selected as the donor, primarily due to his close proximity to the recipient, which allowed for safe and efficient transport, better on-site staff coordination, and enhanced safety for both animals during anesthesia.

On the following day (Day 3), both tapirs underwent general anesthesia, beginning with the donor. The male donor tapir was anesthetized using a combination of 4 mg medetomidine, 40 mg butorphanol, and 60 mg ketamine, mixed into a single dart and administered intramuscularly via a remote dart delivery system (DAN-INJECT Aps, Borkop, Denmark). The tapir was then intubated, and the anesthesia was maintained with 2% isoflurane. Two units of fresh whole blood (300 mL per unit) were collected from the cephalic and saphenous veins using an aseptic technique. Reversal agents, including 120 mg naltrexone and 20 mg atipamezole, were administered intramuscularly after all procedures were completed, requiring approximately one hour. The donor received iron and vitamin C supplementation for two weeks post-procedure. No complications were observed during or after the blood donation procedure.

The recipient tapir was anesthetized using the same protocol. Prophylactic medication, including 60 mg of chlorpheniramine malate and 25 mg of dexamethasone sodium phosphate, was administered to reduce the risk of transfusion reactions. Sixteen-gauge intravenous catheters were placed into cephalic and saphenous veins. Fresh whole blood was transfused at a rate of one drop per second for the first 10 min, followed by 10–20 mL/kg/h. Vital signs and body temperature were closely monitored throughout the transfusion procedure. After the transfusion, the recipient received the same reversal as the donor. The animal regained consciousness within 15 min after antidote administration. Supportive medical management during anesthesia included intravenous fluid (0.9% normal saline solution at 5 mL/kg/h), a gastrointestinal stimulant (metoclopramide, 0.25 mg/kg IV), deworming (ivermectin, 0.4 mg/kg IM), vitamin B complex (0.5 mL/10 kg IM), iron dextran (0.5 mL/10 kg IM), and 20 mL of 50% glucose administered slowly via intravenous injection.

Treatment strategies included the administration of antibiotics (sulfonamides–trimethoprim, 20 mg/kg IM once daily for 7 days) and a non-steroidal anti-inflammatory drug (flunixin meglumine, 1 mg/kg IM once daily for 3 days). Oral supportive therapy included daily supplementation with folic acid for one month and hematinic support (FBC^®^, five tablets), vitamin B complex (five tablets), vitamin C (five tablets), and a liver supplement (silymarin^®^, 140 mg/tablet, five tablets). Supportive management also involved dietary enrichment with fresh leaves, shoots, and branches to promote appetite and recovery. The animal’s overall condition began to improve by Day 7, with urine color returning to normal. The second and third doses of diminazene aceturate were administered two weeks apart. A follow-up blood examination on Day 14 showed an increase in packed cell volume (PCV) from 9% to 14%, and further improvement to 24% by Day 28. By Day 42, PCV had returned to the normal range (33%), and no infection relapse was observed.

Following the diagnosis of severe anemia in one individual, a screening of Malayan tapirs in the same facility was conducted. The facility housed a total of 17 tapirs. Initially, blood samples were collected from five individuals considered for blood donation, all of which were negative for hemoparasites on blood smears. Subsequently, blood was collected from four additional tapirs based on positive reinforcement without anesthesia, bringing the total number of sampled animals to 10 (1 clinically ill, 5 during blood donation screening, and 4 for disease surveillance). PCR targeting the 18S rRNA gene revealed an 80% apparent prevalence of piroplasmid infection among the sampled animals (8/10). Notably, three tapirs that had previously tested negative on blood smears were later found to be positive by PCR, whereas the tapir selected as a blood donor remained negative on both smear and PCR. As the other tapirs did not exhibit clinical signs, no treatment was initiated; however, routine health monitoring was scheduled for every six months.

**Figure 3:**
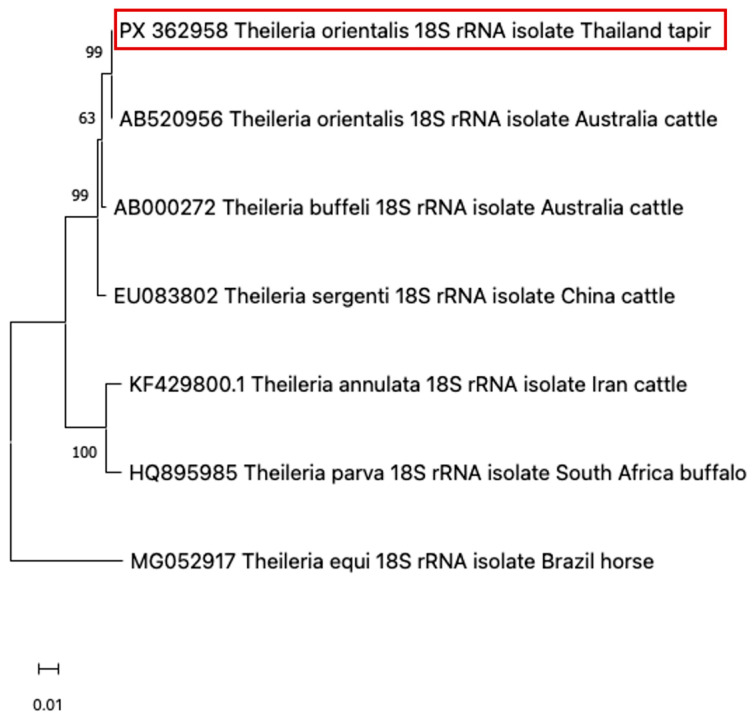
Based on the 18S rRNA locus, a phylogenetic dendrogram illustrated that the sample from the tapir in this case (red-colored box) was closely related to *T. orientalis.*

Due to the morphological similarity among piroplasm species, molecular testing is required for accurate species identification. For this purpose, blood samples were submitted to the Faculty of Veterinary Medicine, Kasetsart University, for molecular diagnosis. Genomic DNA was extracted from whole blood collected during the clinical period using a modified phenol–chloroform method [13]. Universal primers for piroplasm were used to amplify the 18S rRNA region—BAB1w (5′-GAA CCT GGT TGA TCC TGC CAG T–3′) and BAB2w (5′-GAT CCT TCT GCA GGT TCA CCT A–3′)—under conditions previously described [14]. The Platinum™ Hot Start PCR Master Mix (Thermo Fisher, Waltham, MA, USA) was used in each reaction according to the manufacturer’s protocol. The positive PCR product of the correct size was purified using a FavorPrep™ GEL/PCR purification Kit (Favorgen Biotech Co., Pingtung, Taiwan) according to the manufacturer’s instructions. The purified amplicons were sequenced using a BigDye® Cycle Sequencing Kit (Applied Biosystems, Foster City, CA, USA) and an ABI PRISM 2120 Automated DNA Sequencer (Ibis Biosciences, Carlsbad, CA, USA). The resulting nucleotide sequences (GenBank Accession No. PX362958) were aligned and compared with reference sequences that are available in GENBANK using MUSCLE in MEGA version 11 [15] with standard settings and trimmed. A maximum likelihood (ML) phylogenetic tree was constructed for piroplasm species identification. Before constructing the ML tree, a best-fit substitution model was selected based on MEGA version 11 recommendations. The Tamura-Nei model with a discrete gamma distribution (T93+G) was used to generate a phylogenetic tree with a thousand bootstrap replicates. The sequence analysis of the 18S rRNA gene revealed that the sample from the tapir in this case report clustered closely with *T. orientalis*, which can be seen as a clear cluster with a high bootstrap support value (Figure 3).

## 3. Discussion

This report documents the first confirmed case of clinical theileriosis with successful treatment in a captive Malayan tapir in Thailand. The only previous account of piroplasm infection in this species dates back to 1972, when trophozoites were observed in blood and spleen smears of a Malayan tapir; however, no clinical signs or further diagnostic investigations were reported [16]. More recently, *Theileria equi* has been detected in South American tapirs in Brazil, though these findings were incidental and unrelated to clinical symptoms [11]. Similarly, a 2020 study reported *T. equi* in 58% of healthy South American tapirs (*n* = 19) [12]. This suggests the potential role of tapirs as asymptomatic carriers or natural reservoirs of *Theileria* spp.

In endemic areas, animals often develop endemic stability, where persistent parasitemia occurs without overt clinical signs [10]. However, stressors such as translocation, environmental changes, pregnancy, or lactation can compromise immune function and precipitate clinical disease. This phenomenon has been described in various ungulate species, including captive reindeer (*Rangifer tarandus*) [17], roan antelope (*Hippotragus equinus*) [18], sable antelope (*Hippotragus niger*), and greater kudu (*Tragelaphus strepsiceros*), all of which exhibited clinical signs such as weakness, jaundice, anemia, hemoglobinuria, and prolonged clotting times association with *Theileria* infections [19].

Vector-borne parasitic infections represent a significant and often under-recognized threat to health and conservation of tapirs in Thailand. These hemoparasites can compromise individual animal welfare and undermine captive breeding and ex situ conservation efforts for this endangered species. Notably, four captive tapirs, comprising three Brazilian tapirs (*Tapirus terrestris*) and one Malayan tapir, have succumbed to *Trypanosoma evansi* infections in another zoo facility within Thailand [20]. Furthermore, two years prior to the present case, a Malayan tapir housed in the same facility exhibited clinical signs consistent with the present case and subsequently died. It was later diagnosed with *Babesia/Theileria* infection; however, no molecular confirmation was obtained at the time (Maikaew, U.; data unpublished). These two separate incidents in captive facilities of Thailand highlight the susceptibility of tapirs to hemoparasitic diseases, which can cause substantial morbidity and mortality if not promptly diagnosed and managed. In the current study, *T. orientalis*—a species typically regarded as non-pathogenic in domestic cattle [21]—was identified in a Malayan tapir that developed clinical disease. This clinical manifestation is likely attributable to immunosuppression induced by physiological stress associated with recent parturition and lactation, illustrating how stressors can precipitate progression from asymptomatic infection to overt pathology.

Blood smear examination was used as a rapid initial screening method; however, it may fail to identify low-level parasitemia, particularly in subclinical or mild infections. In the present case report, ten tapirs were sampled, and only the individual exhibiting clinical signs tested positive on blood smear, whereas the other seven tapirs were positive only by PCR. These findings highlight the importance of molecular diagnosis and support the use of PCR-based screening in combination with routine blood smear examination to accurately monitor infection status, prevent disease progression, and support the long-term success of tapir conservation programs. Notably, individuals positive for piroplasmid infection on PCR remained asymptomatic and did not require treatment. Consequently, routine health monitoring is essential, as *T. orientalis* may persist sub-clinically in otherwise healthy individuals, with the potential to cause overt disease under immunocompromised conditions.

In the current case, treatment with diminazene aceturate alongside supportive care, including blood transfusion, resulted in marked clinical improvement and normalization of hematological values over a 42-day period. Although diminazene aceturate was effective in this case, it is not generally regarded as the first-line treatment for theileriosis. The drug is primarily indicated for the treatment of babesiosis and trypanosomiasis, and its efficacy against *Theileria* spp. is considered variable [9,22]. In contrast, buparvaquone has been shown to be highly effective against *T. annulata* and *T. parva*, the primary causative agents of bovine theileriosis [23,24]. However, due to the limited availability of buparvaquone, diminazene aceturate was selected in this case. It is also noted that the dosage of diminazene aceturate used was lower than the standard 3.5 mg/kg recommended for cattle [25], owing to the debilitating condition of the tapir. Despite this, the reduced dose was well tolerated and clinically effective. Adverse effects of diminazene aceturate, including neurotoxicity, hepatotoxicity, nephrotoxicity, and local injection site reactions, have been reported in domestic animals [26,27]. In this case, no overt adverse effects were observed following the administration of three doses at two-week intervals. This successful outcome suggests that diminazene aceturate may offer a therapeutic benefit in treating *Theileria* infections in certain non-domestic species, although further studies are needed to confirm its efficacy and safety in tapirs and other wildlife species.

*Theileria* spp. are tick-borne protozoan parasites with a complex life cycle involving both vertebrate hosts and tick vectors [6,28]. Vector control is therefore a key aspect of disease management, particularly in zoological and conservation settings, where multiple species are kept in close proximity under semi-confined conditions. At the time of clinical presentation, the affected tapir was infested with ticks, emphasizing the importance of implementing comprehensive tick control strategies. These may include habitat modification, acaricide application, and regular physical inspections. Currently, all tapirs in the facility are enrolled in a routine tick prevention and hematological examination every 4–6 months. Since the implementation of this protocol, no further *Theileria* infections or relapses have been detected. This case highlights the importance of vigilant vector control, stress management, and early therapeutic intervention in mitigating the impact of *Theileria* infection in wildlife species under human care.

## 4. Conclusions

This case report documents the first clinical diagnosis and successful management of *T. orientalis* infection in a captive Malayan tapir in Thailand. The tapir presented with severe hemolytic anemia, confirmed via microscopy and PCR. Treatment with diminazene aceturate and blood transfusion resulted in full recovery, with normalization of hematological parameters by Day 42. The high prevalence of asymptomatic tapirs in the same facility underscores their potential role as reservoir hosts. Importantly, stress-related immunosuppression likely precipitated clinical disease in the affected individual. This case emphasizes the critical need to integrate hemoparasitic disease surveillance into routine monitoring programs, particularly in ex situ conservation settings where interspecies contact may facilitate transmission. Implementing integrated vector control, routine hematologic monitoring, and timely intervention is essential not only for individual animal welfare but also for the long-term sustainability of threatened species conservation efforts.

## Data Availability

The original contributions presented in this case are included in the article. Further inquiries can be directed to the corresponding author.
